# Mindfulness in Sexual Activity, Sexual Satisfaction and Erotic Fantasies in a Non-Clinical Sample

**DOI:** 10.3390/ijerph18031161

**Published:** 2021-01-28

**Authors:** Laura C. Sánchez-Sánchez, María Fernanda Valderrama Rodríguez, José Manuel García-Montes, Cristina Petisco-Rodríguez, Rubén Fernández-García

**Affiliations:** 1 Department of Evolutionary and Educational Psychology, Faculty of Science Education and Sport, University of Granada, Calle Santander, Nº 1, 52071 Melilla, Spain; lcsanchezsa@ugr.es; 2 Department of Psychology, University of Almería, Carretera Sacramento S/N, La Cañada de San Urbano, 04120 Almería, Spain; mafe_707@hotmail.com (M.F.V.R.); jgmontes@ual.es (J.M.G.-M.); 3 Faculty of Education, Pontifical University of Salamanca, Calle Henry Collet, 52-70, 37007 Salamanca, Spain; 4 Department of Nursing, Physiotherapy and Medicine, University of Almería, Carretera Sacramento S/N, La Cañada de San Urbano, 04120 Almería, Spain; rubenfer@ual.es

**Keywords:** mindfulness, meditation, sexual satisfaction, sexual activity, sexual/erotic fantasies

## Abstract

The goal of this study is to better understand the relation between the practice of Mindfulness and the sexual activity, sexual satisfaction and erotic fantasies of Spanish-speaking participants. This research focuses on the comparison between people who practice Mindfulness versus naïve people, and explores the practice of Mindfulness and its relation with the following variables about sexuality: body awareness and bodily dissociation, personal sexual satisfaction, partner and relationship-related satisfaction, desire, subjective sexual arousal, genital arousal, orgasm, pain, attitudes towards sexual fantasies and types of sexual fantasies. The sample consisted of 106 selected adults, 32 men and 74 women, who completed six measures on an online survey platform: (a) Mindfulness Attention Awareness Scale (MAAS), (b) Scale of Body Connection (SBC), (c) New Sexual Satisfaction Scale (NSSS), (d) Scale of Sexual Activity in Women (SSA-W) and Men (SSA-M), (e) Hurlbert Index of Sexual Fantasy (HISF), (f) Wilson’s Sex Fantasy Questionnaire. In the MAAS, Body Awareness subscale (SBC), NSSS, SSA-W and SSA-M, HISF and intimate fantasies subscale (Wilson’s questionnaire), people in the Mindfulness condition showed higher scores and these differences were statistically significant. These results may have relevant implications in the sexuality of clinical and non-clinical samples.

## 1. Introduction

Mindfulness is a translation of the Pali word “Sati”, which does not have a literal translation in Spanish [1] but refers to the ability to become aware of the present. Practicing mindfulness makes individuals develop their awareness of either sensation, thought or activity (internal or external), without judgment and with radical acceptance.

Many authors have defined the concept of Mindfulness [2,3,4,5]. Jon Kabat-Zinn (1994) defined Mindfulness as a form of “paying attention in a particular way: on purpose, in the present moment, and non-judgmentally” (p.4) [6]. The results of the practice of Mindfulness have been researched in several fields, including health, education and work or business [1]. In the field of mental health, the efficiency of Mindfulness has been demonstrated in people diagnosed with depression [7], in the prevention of depression relapses [8], in somatization disorder [9], suicide prevention [10], general anxiety [11], addictions [12], personality disorders [13], prevention of eating disorders [14], among others. However, research into the practice of Mindfulness in relation to sexuality is limited, both in the clinical [15] and, especially, in non-clinical population [16].

In recent years, research on functional and anatomical changes in the brain that appear as result of practicing mindfulness, as well as relaxation practices, have made extensive progress. All of them coincide with changes in the anterior cingulate cortex and the insula related with body conscience, and the prefrontal cortex linked to emotional regulation [17]. This fact is of vital importance, because it influences the improvement of attention capacity, emotional regulation and perception of body sensations. In fact, the attention to the body sensations is of the utmost importance for the sexual field, because the identification of physiological reactions to sexual activity might influence the experience of a more aware, satisfactory sexuality.

In 2007, Brotto and Heiman developed a study on Mindfulness and sexuality [18]. In this research, they found that being aware of sensory stimuli improved the ability to identify them, and produces a more heightened response. The authors also identified that women who had been unable to pay attention to their arousal and sexual response after undergoing surgery for a hysterectomy, at the time of practicing a self-observational exercise based on Mindfulness, were able to pay attention to their sexual response. In fact, Dr. Lori Brotto and her colleagues have carried out a series of studies on the application of mindfulness for sexual dysfunction, principally in women [19,20,21,22,23,24,25,26,27,28,29,30].

In 2013, Lazaridou and Kalogianni explored the hypothesis of the correlation between attention and sexuality in healthy people [31]. Their research was carried out with 51 participants who had been practicing yoga or meditation, 21 participants who had been doing sport for more than 10 years and 145 participants who had not done any of these activities. The results indicated that the subscales of Mindfulness positively correlated with sexual motivation and sexual awareness. Specifically, people who had been practicing meditation or yoga showed higher levels in novelty-seeking compared to those who had been doing sport.

However, very little research has investigated whether dispositional or practice in mindfulness contributes to positive sexual outcomes in non-clinical samples [32,33,34,35]. The majority of these researches (clinical and non-clinical) were carried out only with women and to best we know no research has included sexual fantasies as important variable to consider. 

One of the most important achievements in Mindfulness practice is the generalization of its learning in different areas (family, society, academic, professional and the affective field, among others). In this way, possible repetition of a problem is easily faced; meaning that when facing a new unsatisfactory situation, a person will be able to use the strategies they have learned to cope with situations in a more effective and self-compassionate way.

If people focus their attention on sexual activity, then their senses and perception of corporal sensations will be heightened and much more vivid than when we are distracted. As soon as they pay attention to their breathing, what they touch, the movements that they make, their corporal changes and to all their sexual experience, then sexual activity improves because there are no goals, demands, preferences, habits or aversions [36]. They simply enjoy the nowness with an open-minded attitude, which means with unprecedented attention, as if we were living the experience for the first time.

According to earlier data and bearing in mind the limited information on this topic, the aims of this research are to identify those areas of sexual activity that can change with the continued practice of Mindfulness, as well as to contribute with more data for future research with larger samples, in a clinical and non-clinical way. Therefore, the main goal of this research is to explore and describe the possible relation of the practice of Mindfulness to sexual activity, sexual satisfaction and erotic fantasies.

The specific objectives of this research are: (1) to describe the social-demographic characteristics of the participants related to the practice of Mindfulness and the sexual variable; (2) to explore the relation of the practice of Mindfulness to body conscience (perception of corporal sensations, processes and actions); (3) to describe the effect of the practice of Mindfulness in sexual satisfaction and activity; (4) to know the possible relation of the practice of Mindfulness to attitudes towards sexual fantasies and to different kinds of sexual fantasies. 

## 2. Materials and Methods 

### 2.1. Participants

The participants were selected by convenience sampling. A snowball method was used to select the sample and to distribute questionnaires on online social networks (Facebook and Linkedin) for the control group, encouraging participants to share the link to the survey webpage among their acquaintances. The same method was used for the Mindfulness group. The distribution of questionnaires was also sent directly to students via email from the institutions that were contacted and had chosen voluntarily to participate in this research: The University of Almería, the University of Zaragoza, the Catholic University of Colombia and Médicos del Mundo NGO. The general inclusion criteria were to be of legal age (over 18), in the countries where the study was conducted, and not to have a clinical problem (mental disorder or any pathology that may affect sexuality). The specific inclusion criteria for the group of Mindfulness practitioners was that they had been practicing for at least 1 to 5 months and practicing for at least 1–2 h a week. 156 people responded to the survey, 50 of whom did not fulfill the inclusion criteria or did not complete the survey and so were discarded. The sample finally consisted of 106 adults (32 men and 72 women) between 18 and 54 years old (M = 28, SD = 8.51). Participants who practiced Mindfulness were 47.2% and 52.8% had never practiced any kind of Mindfulness activity before this study. Of the total sample, 43.4% of them were Spanish and 56.6% were Colombian; 41.3% practiced some religion and 54.7% did not practice any. 

Regarding the type of meditation, out of the 24.5% of the participants who were practicing meditation at the time of answering the study measures: 14 people were practicing vipassana meditation, 3 metta meditation, 4 transcendental meditation, 6 zazen meditation, 7 Buddhist meditation, 6 mantra meditation, 2 Sufi meditation, 3 Dzoguen meditation, 2 Osho meditation, 19 were practicing yoga, 1 person was practicing Thai chi and 3 people were practicing Chi kung. 

### 2.2. Procedure

The application of the scales and questions regarding social-demographic data were displayed on an online survey platform (SurveyMonkey). The link to this survey was sent by email to different organizations dedicated to Mindfulness research and teaching, 5 organizations in Spain and 5 in Colombia. The survey was also shared on social networks and at the Universities of Almería and Zaragoza, which offer Mindfulness postgrad training. Only two organizations answered affirmatively: Médicos del Mundo NGO and Catholic University of Colombia plus the Universities of Almería and Zaragoza. On the invitation to take part, the participants were explained all research objectives and survey rules. Informed online consent was obtained from all individual participants included in this study. Participation was entirely voluntary, and the anonymity and confidentiality of answers were guaranteed to be used only for the research purposes. All procedures performed with human participants were in accordance with the ethical standards of the Bioethics Committee of Human Research of the University of Almería (UALBIO2018/025) and with the 1964 Helsinki declaration and its later amendments or comparable ethical standards.

### 2.3. Measures

Before the implementation of the scales, some social-demographic questions were asked (gender, age, nationality, marital status, level of education, profession, sexual orientation, if they were in a relationship, if they had sexual relations and, finally, if they had children), as well as some questions about religious behavior: being identified or not as a religious person or as a spiritual person, previous practice of Mindfulness, frequency of practice, types of meditation and areas where improvement has been recognized since the practice of Mindfulness.

#### 2.3.1. Mindfulness Attention Awareness Scale (MAAS)

This is a simple scale with a quick, easy implementation which evaluates the person’s ability to be attentive and conscious of the experience at the moment, in their daily lives, with only one score. It consists of 15 items which are scored using a Likert scale on a range from 1 (almost always) to 6 (almost never), higher scores mean a higher state of Mindfulness. The psychometrical analysis of the Spanish version of MAAS showed good properties in validity, such as in reliability (Cronbach’s alpha coefficient = 0.89) [37].

#### 2.3.2. Scale of Body Connection (SBC)

This scale consists of 20 items which are scored with a Likert scale on a range between 0 (never) and 4 (always). There are two independent factors included in this scale, the first one being body awareness, which assesses the conscious attention to sensory signs that indicate the body state (e.g., tension, anxiety, relaxation). The second is bodily dissociation, which measures corporal disconnection or separation of emotional experiences [38]. This subscale is understood as the avoidance of the internal experience and the reaction to emotions [39]. Internal consistence was calculated for the Spanish version of the scale for the two independent factors. The subscale body awareness showed Cronbach’s alpha coefficient of 0.86. Cronbach’s alpha coefficient for bodily dissociation factor was 0.62.

#### 2.3.3. New Sexual Satisfaction Scale (NSSS)

This survey consists of 20 questions with a multidimensional approach, in which three factors are differentiated: Ego-centered satisfaction, partner-centered satisfaction and sexual activity-centered satisfaction. The score is obtained by a Likert scale on a range of 5 points, from 1 (dissatisfied) to 5 (fully satisfied) for each item. The scale validated to Spanish showed a coefficient Cronbach’s α of 0.93 [40]. Higher scores indicate higher sexual satisfaction. 

#### 2.3.4. Scale of Sexual Activity in Women (SSA-W) and Scale of Sexual Activity in Men (SSA-M)

This scale assesses sexual activity in women and men separately [41,42,43]. It contains 8 items in the female version and 10 items in the male version. This scores desire, subjective sexual arousal, lubrication/ejaculation, orgasm, pain and satisfaction. It is scored by a Likert scale on a range of 5 points, from 1 (Never) to 5 (Almost always- Always). The items 4, 5 y 7 are scored conversely in both versions (SSA- W) and (SSA- M). The cut-off point is a score of 26.55 or lower. 

#### 2.3.5. Hurlbert Index of Sexual Fantasy (HISF)

This scale evaluates the participants’ attitudes towards sex fantasies by 25 items of a Likert scale on a range of 4 points, from 4 (Never) to 0 (Always). The total score is between 0 and 100, and any higher score means higher positive attitudes towards sexual fantasies. In the Spanish version, the scale contains two subscales, one positive and one negative, and Cronbach’ coefficient is of 0.85 and 0.83, respectively [44,45]. 

#### 2.3.6. Wilson’s Sex Fantasy Questionnaire

The Spanish version of Wilson’s Sex Fantasy Questionnaire was developed by Sierra, Ortega, Martín-Ortiz and Vera-Villarroel [46], and subsequently, Sierra, Ortega and Zubeidat [47]. This scale consists of 24 items which are divided into 4 subscales: (a) exploratory sex fantasies, (b) intimate sexual fantasies, (c) impersonal sexual fantasies and (d) sadomasochistic sexual fantasies. The scale is assessed by a Likert scale on a range from 0, which means never, to3, which means often. The total score is between 0 and 18. The scale has a total internal consistency of 0.90, which fluctuates between 0.66 and 0.79 for its different subscales.

### 2.4. Data Analyses

SPSS for Windows, 24.0 version (IBM Corporation, Armonk, NY, USA) [48], was used to analyze the data obtained. Firstly, with respect to social-demographic questions, descriptive analyses were carried out. Secondly, Cronbach’s α coefficient was calculated for each applied scale in this Spanish-speaking sample (see Supplementary Material). 

Before the analysis, the normality of the data was calculated with Kolmogorov-Smirnov test. MAAS scale and the subscales of the Scale of Body Connection (SBC): body awareness and bodily dissociation kept a normal distribution, but NSSS, SSA-W, SSA-M, Hulbert Index and the subscales of the Wilson Questionnaire did not show a normal distribution. The nonparametric U of Mann-Whitney test was used to compare independent samples, due to the majority of the variables not showing normal distribution, all these subscales are ordinal, and the sample was not random.

So, thirdly, effects of meditation practice in mindfulness, body connection and sexual variables were analyzed with an independent-samples Mann-Whitney test on both conditions. Cohen’s d was used as a measure of effect size for all between-group comparisons [49]. Finally, exploratory analyses also examined bivariate correlations among separate facets of mindfulness and the variables of interest.

## 3. Results

### 3.1. Social-Demographic Characteristics of the Participants

Finally, 106 participants, 74 women and 32 men, took part in this study (see Table 1). With respect to participants’ professions: 65.1% of the sample had career connected to the health profession and were principally psychologists; 13.2% were teachers; 10.4% were students; 2.8% were technologists; 1.9% were engineers; 1.9% were managers of companies; 1.9% were professionals related to social sciences and 0.9% were musicians. In relation to meditation practice: 47.2% of the sample had practiced Mindfulness, of which 15.1% had been practicing it between 1 and 5 months, 11.3% between 6 and 11 months, 8.5% between 12 and 23 months, 2.8% between 24 and 35 months and 9.4% from 36 months ago. The rest of the sample, 52.8%, had never practiced Mindfulness when they participated in answering the survey.

**Figure 1:**
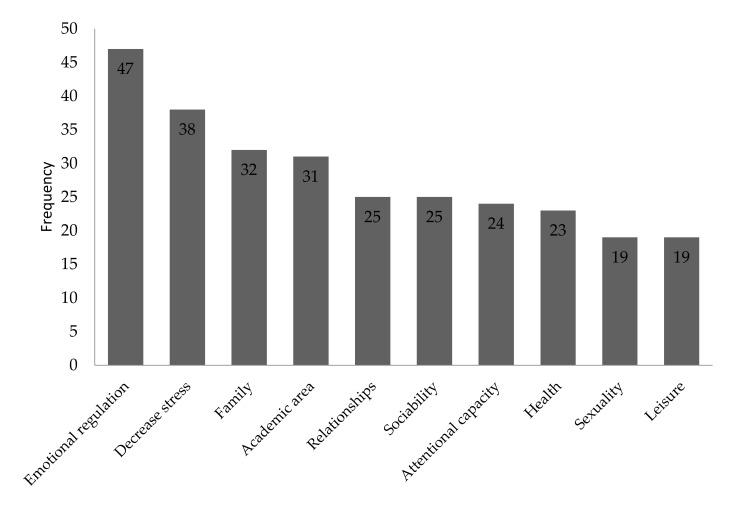
Increased areas with the practice of Mindfulness according to participants.

Figure 1 shows increased areas with Mindfulness practice. The highest increase is in emotional regulation; secondly, stress decreased and finally the improvement in the familiar relations. The fields with a lower score were: (a) improvement in physical health, (b) the sexual field and (c) enjoyment of leisure and free time (see Figure 1). 

### 3.2. Mindfulness and Body Connection

In the MAAS, the score was higher in people who practiced Mindfulness than in those who did not (Mann-Whitney U test = 863.0, *p <* 0.001); this result showed a moderate effect size (d = 0.69, r = 0.32). In relation to Scale of Body Connection, the subscale of body awareness showed a higher score in people who practiced Mindfulness than in people who did not (Mann-Whitney U test = 921.0, *p =* 0.002), and this result showed a moderate size-effect (d = 0.48, r = 0.23). However, bodily dissociation subscale showed a lower score on Mindfulness practicing than on non-practicing, but there were no statistically significant differences between the conditions (Mann-Whitney U test = 1140.5, p = 0.099). 

### 3.3. Differences in Sexual Satisfaction and Activity

In the NSSS, which measures sexual satisfaction, participants who practiced Mindfulness had higher scores than participants who did not (Mann-Whitney U test =1037.0, p=.021), but this result showed a small effect size (d = 0.25, r = 0.12). The comparison between both conditions obtained the same results for the SSA in both genders; SSA-W scale (Mann-Whitney U test = 461.5, *p =* 0.039), which showed a moderate effect size (d = 0.57, r = 0.27) and SSA-M scale (Mann-Whitney U test = 98.0.5, *p =* 0.009) which showed high-moderate effect size (d = 1.06, r = 0.46) (see Table 2). 

### 3.4. Differences in the Attitud towards Sexual Fantasies and the Frequency of the Severaltypes of Them

In relation to the Hurlbert Index of Sexual Fantasy (HISF), scores indicated that people who practice Mindfulness had more positive attitudes towards sex fantasies (Mann-Whitney U test = 1061.0, *p* = 0.032), and a low-moderate effect size (d = 0.45, r = 0.22). Finally, Wilson’s Sex Fantasy Questionnaire results indicated that in all subscales people who practice Mindfulness had higher scores, but there were only statistically significant differences in the intimate subscale (Mann-Whitney U test = 836.5, *p* < 0.001), which had a moderate size-effect (d = 0.69, r = 0.32) (see Table 2).

With respect to the gender differences in the questionnaires, there were only differences in the Wilson Sex Fantasies Questionnaire on the intimate fantasies subscale (Mann-Whitney U test = 864, *p* = 0.027), being high in men than in women and with a low-moderate effect size (d = 0.48, r = 0.23).

### 3.5. Correlations between Variables in the Whole Sample

After applying a correlation analysis, it was appreciated that the MAAS scale had a positive and statistically significant correlation with following subscales: body awareness (r = 0.406, *p* < 0.001), sexual satisfaction measured by NSSS (r = 0.495, *p* < 0.001), and the Hurlbert Index of Sexual Fantasy (HISF) (r = 0.267, *p* < 0.001). MAAS scale had a negative correlation with the subscale of bodily dissociation (r = −0.396, *p* < 0.001), which means when one increases the other one proportionally decreases. Every variable was analyzed with the Spearman test, however, in Table 3 and Table 4 only statistically significant results are shown (see Table 3 and Table 4).

The variable “quantity of Mindfulness practice” had a positive correlation with the MAAS scale (r = 0.319, *p* = 0.001), sexual satisfaction by NSSS (r = 0.222, *p* = 0.022) and body awareness (r = 0.341, *p* < 0.001), as it is showed in the Table 4.

## 4. Discussion

The research achieved its main goal: to know and describe the relation of the practice of Mindfulness to sexual activity, sexual satisfaction and erotic fantasies. The results of this research indicate that there are three fields in which participants who practice Mindfulness showed a more positive influence of the meditation: (a) emotional regulation, (b) stress decreased, (c) improvement of familiar relationships. One of the lowest influences of the practice of Mindfulness is in the field of sexuality, however the results of the applied measures on participants who practice Mindfulness are higher than in participants who do not. This fact might be explained, firstly, because there are few publications explaining the effects of the practice of Mindfulness in the field of sexuality. Scientific literature on Mindfulness publishes results of other areas which have been studied extensively, such as emotional regulation. As a result, people focus on the known benefits of Mindfulness and ignore the possible effects of its practice in other areas. Secondly, people who identify with an improvement in this area, they prefer to focus on other benefits, such as decreasing stress. It is worth mentioning that people who learn about Mindfulness do not apply it to the sexual field or get in contact with professionals who could guide them in this implementation. Common thought is that Mindfulness does not have any significant effect on the sexual area. Another explanation can be that Mindfulness improves emotional regulation and this plays a role as mediator in enhancing sexuality [50]. 

In the same way, results indicate that people who practice Mindfulness tend to pay more attention to sensory signs that indicate a corporal state, as the results in body awareness show. In a previous study it was found that being aware of sensory stimulus improves the ability to identify them and provide a significant answer [51]. Likewise, the scores of participants who practice Mindfulness are lower than participants who do not practice it on the subscale of bodily dissociation [52]. This means that people who do not practice Mindfulness probably show a higher level of avoidance of internal experience and a higher difficulty in identifying, expressing and paying attention to body sensations and emotions.

The scales of NSSS, SSA-W and SSA-M, which measure sexual satisfaction, show higher scores in those participants who practice Mindfulness than in those participants who do not. The highest score was in ego-centered satisfaction, partner-centered satisfaction and sexual activity-centered satisfaction, desire, subjective sexual arousal, lubrication/ejaculation and orgasm. Sexual satisfaction is defined by interpersonal and intrapersonal fields much more than social-demographic aspects [53]. In 1987sexual satisfaction (with respect to women) was defined as: “a subjective evaluation of the degree to which a woman is satisfied with their sex life” (p. 234) [54]. Sexual satisfaction relates to: (a) orgasm frequency, (b) sexual activity frequency, (c) intimacy level, (d) partner communication (e) desire and (f) emotional expression. According to previous information, in this sample, Mindfulness participants reached higher scores in sexual and partner satisfaction, possibly because Mindfulness is known to favor emotional regulation, emotional expression and body consciousness [50]. The expression of emotions is important in a relationship to solve conflict, express needs and discomfort and to positively reinforce each other. Sexual satisfaction relates to body consciousness as well, because this implies paying attention to sensory signs that indicate a corporal state, in this case sexual activity. 

People who were practicing Mindfulness reached higher scores on the Hurlbert Index of Sexual Fantasy (HISF) scale, meaning they had more positive attitudes towards sexual fantasies. Consistent with other authors, this is an indicator of good sexual health [55], higher levels of sexual desire [56] and constitutes a good indicator of sexual functioning in older women [57]. The initial research made by Sánchez-Sánchez, Luciano and Barnes-Holmes (2009) showed that the effort to suppress an exciting thought brings this more strongly by a rebound effect [58]. This is related to the results obtained and explains the score differences between people who practice Mindfulness and those who do not. The practice of Mindfulness supposes a higher attentional capacity and ability to live in the moment without value judgments, this situation makes it easier to accept sexual fantasies and sexual thoughts and live them in an open-minded way. 

In the Wilson’s Sex Fantasy Questionnaire, participants who practiced Mindfulness had higher scores in all subscales (exploratory, intimate, impersonal and sadomasochist). The subscales with the highest means in Mindfulness’ practitioners were the intimate and sadomasochist subscales. The first subscale is related to deep pleasure in a romantic commitment or with a limited number of sexual partners [59], where it is characteristic to follow these actions: getting undressed, fellatio, having sex in another place than the bedroom. With respect to gender differences, men scored high in this subscale. Although differences between gender are not usually found in this subscale, the result is consistent with some previous research [60]. The second subscale is associated with submission behavior or a pain provocation during sexual arousal and includes actions such as: being flogged, being tied up and to tie someone up, among others. This result is consistent with the Hurlbert scale outcomes, due to participants showing a more open mind to experimenting sexual fantasies and also connects to some aspects that Mindfulness potentiates; such as observation and acceptance of thoughts, because practitioners do not usually identify with these thoughts. This component may have an influence on participants assuming that their fantasies are only thoughts and they can live them without guilt, different from people who feel identified with their fantasies and believe that only thinking about something means that they are doing it in reality.

These results indicate that the sample, in general, has positive attitudes towards sexual fantasies, which means that they live them in a pleasant and acceptable way. These fantasies act like predictors of higher levels of sexual desire, sexual excitement and sexual satisfaction. Lazaridou and Kalogianni (2013) indicated that Mindfulness participants show the highest levels on the innovation search [31]. This fact could be related with the highest scores on exploratory fantasies in comparison with people who have not practiced Mindfulness.

### Limitations and Future Research

The main limitation of this research was to be able to access the sample. Even though a search and contact with the main organizations related to the practice of Mindfulness in Spain and Colombia was carried out, some of them were not interested in supporting this research. During diffusion of research, some Mindfulness practitioners felt uncomfortable that evaluation was about the field of sexuality, so they preferred not to participate in the research. For future research, expanding the sample size is recommended, in order to be able to generalize the results for the general population. Another limitation was the scarce research that had been carried out of the practice of Mindfulness and sexuality in non-clinical samples. This situation does not facilitate a more conscientious analysis of the obtained results and, for this reason, more research into this area is necessary.

The results obtained in this research pave the way to continue the search for the influence that Mindfulness has on the practice of sexual activity. Although this research presents multiple variables about sexuality, it is recommended that future research studies each topic more extensively using a larger sample. One the one hand, it is of great importance to continue research with clinical samples, where the main goal is to explore the introduction of the practice of Mindfulness in sexual therapy alone and with a partner. In fact, this kind of research is beginning to be developed [15,16]. On the other hand, to increase research with non-clinical samples, to know in depth the dimensions of sexuality which could possibly be influenced by the practice of Mindfulness and not only those variables investigated in this research.

## 5. Conclusions

This research has explored the practice of Mindfulness and its relation to sexual activity and the results indicate that the practice of Mindfulness improves sexual health and sexual satisfaction. It also shows that Mindfulness participants have a greater tendency to pay attention to corporal sensations and sexual stimulus, which implies that they were freer of judgment and self-criticism related to carrying out sexual performance, which results in an enhancement in the field of sexuality, and subsequently in their quality of life. Although the results obtained in this research are only exploratory, they are an important contribution to the field of Sexology and to the field of the implementation of Mindfulness. It invites researchers to develop new strategies in clinical and non-clinical populations, including sexual fantasies. Mindfulness is presented as an alternative skill with implications in different dimensions of sexuality and significant results in the promotion of sexual health.

## Figures and Tables

**Table 1:** Participants’ social-demographic characteristics.

Variable	Response Options	Percentage of Participants	N
Sex	Male	30.2%	32
	Female	69.8%	74
Nationality	Spanish	43.4%	46
	Colombian	56.6%	60
Level of studies	Primary studies	0%	0
	Secondary studies	5.7%	6
	Higher education	94.3%	100
Sexual orientation	Heterosexual	85.8%	91
	Homosexual	7.5%	8
	Bisexual	6.6%	7
	Others	0%	0
Practicing of some religion	Yes	41.3%	46
	No	54.7%	58
The participant identifies	Yes	74.5%	79
himself/herself as a spiritual person	No	25.5%	27
Practicing meditation	Yes	24.5%	26
	No	75.5%	80
Practicing Mindfulness	Yes	47.2%	50
	No	52.8%	56

**Table 2:** Mann-Whitney U test, means and standard deviations of applied measures, comparisons between Mindfulness practitioners and non-practitioners.

	Mindfulness Practitioners	No Mindfulness Practitioners	*U de M-W, p*
**Mindful Attention Awareness Scale (MAAS)**	*M =* 67.54, DT = 11.61	*M =* 59.34, DT = 11.94	863 (0.001) **
**Scale of Body Connection (SBC)**			
Body Awareness	*M =* 33.16, DT = 7.84	*M =* 29.52, DT = 7.33	921 (0.002) *
Bodily Dissociation	*M =* 8.12, DT = 4.59	*M =* 9.54,DT = 4.20	1140.5 (0.099)
**New Sexual Satisfaction Scale (** **NSSS)**	*M =* 77.86, DT = 14.43	*M =* 70.08, DT = 15.56	1037 (0.021) *
**Scale of Sexual Activity in Women (SSA-W)**	*M =* 28.93, DT = 3.36	*M =* 26.58, DT = 4.57	461.5 (0.039) *
**SSA in Men** **(SSA-M)**	*M =* 34, DT = 3.12	*M =* 29.73, DT = 5.22	98 (0.009) *
**Hurlbert Index of Sexual Fantasy (HISF)**	*M =* 80.08, DT = 11.29	*M =* 74.55, DT = 12.95	1061 (0.032) *
**Wilson Sex Fantasy Questionnaire**			
Exploratory fantasies	*M =* 7.76, DT = 5.13	*M =* 6.53, DT = 4.41	1188 (0.172)
Intimate fantasies	*M =* 14.98, DT = 3.03	*M =* 12.82, DT = 3.15	836.5 (0.000) **
Impersonal fantasies	*M =* 5.88, DT = 3.86	*M =* 4.91, DT = 3.01	1247.5 (0.331)
Sadomasochistic fantasies	*M =* 8.28, DT = 6.57	*M =* 6.78, DT = 4.52	1247.5 (0.363)

*p < 0.05, ** *p* < 0.001.

**Table 3:** Spearman correlations of the MAAS with subscales of: body awareness, bodily dissociation, sexual satisfaction (NSSS) and the Hurlbert Index of Sexual Fantasy (HISF).

Variable	*r*	*p*
Body Awareness	0.406 **	0.000
Bodily Dissociation	−0.396 **	0.000
NSSS	0.495 **	0.000
HISF	0.267 **	0.006

** The correlation is significant at the level 0.01. N = 106.

**Table 4:** Spearman correlations of the variable quantity of Mindfulness practice with the MAAS, NSSS and body awareness.

Variable	*r*	*p*
MAAS	0.319 **	0.001
NSSS	0.222 *	0.022
Body Awareness	0.341 **	0.000

** The correlation is significant at the level 0.01 * The correlation is significant at the level 0.05. N = 106.

## Data Availability

The data presented in this study are available on request from the corresponding author. The data are not publicly available due to privacy and ethical reasons.

## Supplementary Materials

The following are available online at https://www.mdpi.com/1660-4601/18/3/1161/s1, The Cronbach’s alpha values in each questionnaire for the research sample.
