# Supplementary material for: Mindfulness in Sexual Activity, Sexual Satisfaction and Erotic Fantasies in a Non-Clinical Sample
Source: Int J Environ Res Public Health. 2021 Jan 28;18(3):1161. doi: 10.3390/ijerph18031161 (PMC7908537; doi:10.3390/ijerph18031161)
Supplement: Supplementary file 1 [file ijerph-18-01161-s001.pdf]

The Cronbach's alpha values in each questionnaire for the research sample

The Mindfulness Attention Awareness Scale (MAAS) in this sample had a Cronbach’s α coefficient = 0.89 after analyzing the reliability on the applied tests. In relation to the Scale of Body Connection (SBC), the body awareness subscale showed a Cronbach’s α coefficient = 0.85 and its bodily dissociation subscale showed a Cronbach’s α coefficient = 0.71. In relation to the New Sexual Satisfaction Scale (NSSS), the Cronbach’s α coefficient was 0.95. The Hurlbert Index of Sexual Fantasy (HISF) showed a coefficient α of Cronbach= 0.88 and finally, the Wilson's Sex Fantasy Questionnaire showed a Cronbach’s α coefficient= 0.87. On its subscales: a) exploratory: α = 0.81, b) intimate: α = 0.67, c) impersonal: α = 0.68, d) sadomasochism: α = 0.89.
